# Gonorrhea Treated with the Acetate of Potash

**Published:** 1851-02

**Authors:** 


*Gonorrhœa treated with Acetate of Potash.*—Mr. Hilton has endeavored to arrest gonorrhœa without the aid of cubebs, copaiba, or other means usually adopted. The medicine he employs is acetate of potash in half-drachm doses. The following cases are selected to exhibit the results of this simple treatment:—

The first case refers to a man, aged thirty, a tailor by trade, who, when admitted, July 24th, into Samaritan ward, had been affected with a discharge for a week; the urine was very acid, and the scalding intense. The day after admission, Mr. Hilton prescribed half-drachm doses of acetate of potash to be taken three times a-day. Five days afterwards, the scalding was relieved, the urine being still acid; on the thirteenth day the scalding, though decreased in intensity, continued, and the urine remained acid; the acetate was therefore ordered to be taken every fourth hour. On the sixteenth day the scalding was trifling, and the urine acid; on the eighteenth the urine became alkaline; the scalding had disappeared, and the discharge had completely ceased for some days. The patients are always kept in the house a little time after its cessation, for fear of a relapse; and on the twenty-third day the patient was quite well, after having taken the acetate of potash three times a-day for thirteen days, and every four hours for five days.

The second case is that of a laborer, twenty-seven years of age; he had had a gonorrhœal discharge for three weeks when he applied for admission. The treatment began in the same ward (Samaratan), on the 24th of July, as in the preceding case, and the acetate of potash, in an aqueous menstruum, was likewise ordered to be taken three times a-day. The salt is also given with advantage in camphor mixture, and Mr. Hilton often supplies the patients with the acetate in powder, desiring them to dissolve it in water themselves. Here, however, no very material improvement took place for the first fifteen days, when the scalding became less, and the discharge began to diminish in quantity, the urine being acid. Mr. Hilton now ordered the same doses to be taken every fourth hour. On the nineteenth day the scalding continued, the discharge was less, and the urine slightly alkaline. On the twenty-second the urine became again acid, though the patient was taking the acetate every fourth hour. At this time darrhœa set in, and the patient was precluded from continuing the acetate of potash. On the twenty-eighth day after admission, however, the use of the salt was resumed; it was taken every four hours for eleven days, when the scalding and discharge were entirely subdued. It should, however, be noticed, that even at this period the urine remained somewhat acid.

Mr. Hilton was led to give the acetate of potash a trial in cases of gonorrhœa from the following considerations: The urethra is in this disease in a highly inflammatory state, and the urine very acid; it is clear that the latter fluid in passing over an inflamed surface must tend to irritate it much, and retard the diminution of the inflammation. The acetate of potash, by its tendency to render the urine alkaline, will therefore remove one of the obstacles which lie in the way of the cure. This same salt has likewise most undoubted depurating properties, and its antiphlogistic powers are very remarkable. Its administration, therefore, will have the double advantage of rendering the urine less acid and irritating, and of reducing inflammatory action all through the system, and as a consequence in the urethral mucous membrane.

Mr. Hilton does not deny the modifying properties of copaiba and cubebs, but he considers that the acetate of potash renders great service in those numerons cases where, from many different circumstances, neither copaiba nor cnbebs can be taken. He often uses the latter therapeutical agents when the disease has been subdued by the above-named salt; a few doses of copaiba then complete the cure, and are likely to prevent relapse.—*Lancet*, Nov. 1.

